# What Drives University Students’ Sustained Participation in Volunteering? A Thematic Analysis from the Ecological Systems Theory (EST) Perspective

**DOI:** 10.3390/bs16030471

**Published:** 2026-03-22

**Authors:** Zhanni Luo, Xueqin Peng

**Affiliations:** School of Foreign Languages and Literatures, Chongqing Normal University, Chongqing 401331, China

**Keywords:** volunteer motivation, volunteering, Ecological Systems Theory (EST), thematic analysis

## Abstract

Background: Understanding the drivers of sustained volunteering among university students is crucial, as their continued participation yields significant individual and societal benefits. However, a notable decline in participation underscores the need to investigate the factors that underpin and sustain volunteering motivation. Methods: Using snowball sampling, 15 university students with volunteer experience were recruited for semi-structured interviews. A thematic analysis, guided by the Ecological Systems Theory (EST), was performed, with the mesosystem excluded from the analytical framework due to its indirect and less observable nature in participants’ self-reports. Results: Based on the interview responses, we identified 15 themes across the four systems (microsystem, exosystem, macrosystem, and chronosystem) influencing university students’ participation in volunteering. We further explored the restrictive factors that hinder their participation. To advance the analysis, we introduced a controllability framework (“controllable, partially controllable, minimally controllable”) as an analytical lens. This framework emphasizes that while behaviors are shaped by various factors, behavior change can be most effectively promoted by focusing intervention efforts on those within the immediate control of the individual or relevant actors. Implications: This study demonstrates the EST’s applicability to university students’ volunteering research and provides practical insights for the design of volunteer programs.

## 1. Introduction and Literature Review

### 1.1. Volunteering and the Reduction in Volunteer Participation

Volunteering is defined as a prosocial act performed in service of another person, organization, or group, regardless of whether it aligns with the actor’s self-interest (Breen et al., 2024). It is an act that is performed voluntarily, with no compulsion and no expectation of payment or profit (Sengupta & Al-Khalifa, 2022). Volunteering takes place within large-scale events and various social initiatives, such as community development (Udoma et al., 2024), disaster management (Nahkur et al., 2022), environmental protection (Hung et al., 2022), sporting events (Yoo et al., 2023), education development (Holdsworth & Quinn, 2010), and elderly care service (Lu et al., 2024).

The value of volunteering is threefold, critically enriching the lives of individuals, strengthening institutional missions, and advancing broader societal goals. It promotes personal growth, encourages social responsibility, and enhances employability by helping students develop practical skills (Chen et al., 2025; Hustinx et al., 2010). In addition, volunteering provides psychological benefits such as individuals’ happiness, social connectedness, self-efficacy and perceived autonomy (Alganami & El Keshky, 2025; Jiang et al., 2021), and a stronger sense of meaning in life, which is closely linked to wellbeing (Martela et al., 2018; Martela & Ryan, 2016). For universities, volunteer activities provide a platform through which students can actively engage in practical experiences, applying their knowledge and developing skills beyond the classroom (Chen et al., 2025). For organizations hosting volunteer initiatives, student volunteers help alleviate financial pressures (Allen & Bartle, 2014). Given these substantial benefits, scholars have turned their attention to how to involve more university students in volunteering (Chen et al., 2025).

However, in recent years, the number of volunteers has shown a clear declining trend. According to the United Nations Volunteers (UNV), 44% of the global volunteer workforce ceased volunteering between 2018 and 2021 (Morley et al., 2021). Similarly, in Australia, according to Holtrop et al. (2024), adult participation in volunteering dropped from 36% in 2010 to 27% in 2022. This widespread decline threatens the sustainability of essential community services and the social capital they generate, underscoring an urgent need to investigate the root causes of this decline.

### 1.2. Declining Volunteer Participation and the Importance of Volunteer Motivation

Normah and Lukman (2020) indicate that the decline in student participation in volunteer activities can be attributed to the element of motivation. This highlights the crucial role of motivation, which is defined as the psychological tendency that stimulates and sustains an individual’s behavior, guiding actions toward a specific goal (Luo & Xiong, 2025). As volunteering is a voluntary behavior that requires personal drive and a sense of purpose; without sufficient motivation, students are less likely to initiate participation, or sustain their participation. Consequently, for over half a century, volunteer motivation has been a central topic in volunteer research (Chen et al., 2022; Welty Peachey et al., 2014).

Since university students often constitute the main volunteer workforce, research frequently focuses on understanding the motivation of university student volunteering (Francis, 2011). Research often focuses on the different types of motivations that drive university students, including altruistic, social, career-oriented, and personal development motives (Frisch & Gerrard, 1981; Gage & Thapa, 2012; Morrow-Howell & Mui, 1989). It also examines the individual, social, and contextual factors that shape these motivations, as understanding them is essential for identifying the main determinants of sustained volunteering and for informing strategies to improve retention and foster long-term, sustainable participation (Saksida et al., 2017).

### 1.3. Theories Explaining Volunteer Motivation

Theoretical exploration of volunteer motivation has shown a trend of evolution from simple dualistic divisions toward a multi-level and multi-functional understanding. Early research, influenced by Social Exchange Theory, tended to simplify motivation into a two-dimensional model of altruism and self-serving (Frisch & Gerrard, 1981), which primarily pertained to individuals’ intrinsic psychological tendencies. As theories progressed, scholars began to incorporate the influence of social relationships, forming a three-dimensional model that included altruistic, social and material motivation (Morrow-Howell & Mui, 1989), highlighting interactions between individuals and their immediate environment.

Among these, the Functionalist Theory stands out as the most influential. According to the theory, individuals choose to participate in activities based on whether their perception of an activity aligns with their motivation (Chung et al., 2019). By identifying six motivational functions, such as values, career development, and social interaction, the theory revealed the multifaceted nature of the motivation for participating in volunteer activities (Clary et al., 1998). These functions integrate both individual psychological processes and broader environmental influences.

To understand the motivation for volunteer participation, recent studies have increasingly adopted motivation models, such as Self-Determination Theory (Ryan & Deci, 2000) and Theory of Planned Behavior (Ajzen, 1991). Sheldon et al. (2023) assessed the motivations, commitment, and subjective well-being of 441 food bank volunteers, using motivation scales derived from Self-Determination Theory and Functionalist Theory. Additionally, a study tested if the Theory of Planned Behavior can explain event volunteers’ retention (Lee et al., 2014), and Reuveni and Werner (2015) investigated the factors associated with teenagers’ willingness to volunteer with elderly persons using an expanded model of the Theory of Planned Behavior.

### 1.4. The Application of Ecological Systems Theory (EST) in Volunteer Motivation Research

Existing theories on volunteer motivation have generated important insights, but most focus on fragmented influencing factors. For example, a study on volunteers of the 2023 European Games in Poland only examined individual socio-demographic factors, such as gender, place of residence, professional activity and prior sports volunteering experience in isolation (Rozmiarek et al., 2021). Such fragmentation makes it difficult to understand how influences across different contexts interact to shape and sustain volunteer participation. To address this limitation, we adopt the Ecological Systems Theory (Bronfenbrenner, 1977), which posits that individuals are shaped by complex systems of interrelated environments (Lasekan et al., 2024).

Ecological Systems Theory (EST) conceptualizes human development and behavior as shaped by a set of nested and dynamically interacting environmental systems (Bronfenbrenner, 1977). Rather than viewing volunteer motivation as the product of isolated factors, EST highlights the complex interplay of influences across multiple ecological layers. According to the theory, five interconnected systems jointly structure an individual’s experiences:

- Microsystem: the most proximal and physically distinct setting, such as home, childcare, playground, where an individual resides and engages in face-to-face interactions with others (Bronfenbrenner, 1977).
- Mesosystem: linkages among microsystems, such as interactions between school and family environments, such as transactions and interactions between the immigrant family and their peers (Bronfenbrenner, 1977).
- Exosystem: social structures that indirectly influence individuals, including healthcare, education service systems, support network, and the broader society (Bronfenbrenner, 1977).
- Macrosystem: broader cultural, socioeconomic, educational, legal, and political systems that shape societal norms and expectations (Bronfenbrenner, 1977).
- Chronosystem: changes and transitions in individuals and their environments over time, reflecting the dynamic nature of motivation (Bronfenbrenner, 1979, 1994).

The EST has been widely applied across various fields. Fan and Xie (2025) identified factors influencing pre-service English-as-foreign-language (EFL) teacher anxiety at the personal and macro, exo, meso, micro, and chrono levels. Liu et al. (2022) investigated the factors shaping English teacher buoyancy in online teaching and how teachers shape and exercise buoyancy in their negotiation with different ecological systems in online teaching guided by EST. Teng-Calleja et al. (2024) used the theory to explore organizational and team-level actions perceived as helpful or not helpful by employees in navigating the hybrid work arrangements and further analyzed how these actions impacted employees’ work behaviors and experiences. Additionally, EST-based research examined responses to volunteering as expressed in satisfaction with volunteering and burnout (Kulik, 2007), illustrating the theory’s applicability to volunteer behavior research. Furthermore, Mahmud (2022) examined how school teachers experienced a trauma-informed online professional development and social-emotional learning program intended to improve student outcomes, teacher perceptions, and teacher-student relationships. These cross-domain studies show that EST can systematically analyze multi-level factors in shaping individual attitudes and behaviors.

However, comprehensive qualitative exploration of university students’ volunteer sustained participation using EST’s full five-level systems remains insufficient. To address this gap, this study aims to adopt EST as an integrative framework to systematically identify and understand the factors influencing university students’ volunteer sustained participation, thereby filling a gap in existing research and providing a more comprehensive explanatory perspective.

The selection of the EST is justified by the nature of volunteer participation itself. Volunteer participation emerges from the interplay between personal attributes, interpersonal relationships, organizational structures, and broader sociocultural factors, all of which align with EST’s multi-level analysis. From this perspective, the study can systematically explore these interconnected influences, explaining how university students’ volunteer participation is sustained.

According to EST, the environment can be categorized into five systems (microsystem, mesosystem, exosystem, macrosystem, and chronosystem). Among these, the mesosystem is excluded from the analytical framework of the study. The reason is that the mesosystem represents the interactions and connections between different microsystems (e.g., family-school relations, peer-family interactions), which are often indirect and complex. From the perspective of individual volunteers, it is difficult to accurately observe or articulate these inter-system interactions. Therefore, to ensure the reliability and clarity of the data, this study focuses on the microsystem, exosystem, macrosystem, and chronosystem, which are more directly perceivable and reportable by participants.

Therefore, this study aims to address the following research questions (RQs):

RQ1: What are the microsystem factors that affect university students’ sustained participation in volunteering?

RQ2: What are the combined exosystem and macrosystem factors that affect university students’ sustained participation in volunteering?

RQ3: What are the chronosystem factors that affect university students’ sustained participation in volunteering?

## 2. Methods

### 2.1. Participants and Ethics Considerations

This study employed a thematic analysis approach, in which data collection continued until theme saturation was achieved, namely the point at which new data no longer yielded additional insights or thematic developments. The sample size was determined with reference to the systematic review by Wutich et al. (2024), which suggests that theme saturation is generally attained with a minimum of nine participants and an average of 12 to 13 participants. In line with this guidance, a total of 15 university students with volunteering experience were recruited as participants in the current study.

These 15 participants were drawn from four academic disciplines: Law, Engineering, English Language and Literature, and Teaching English to Speakers of Other Languages (TESOL). Among them, 12 were female and three were male. Six participants were undergraduate students, while nine were enrolled in graduate programs. Due to ethical considerations, specific ages were not collected; however, all participants were generally within the age range of 19 to 24 years, reflecting typical university student demographics. This sample composition provided a diverse yet representative perspective across academic levels and fields.

The participants volunteered in a wide range of activities. These included large-scale sporting events such as marathons, competitions such as Chinese Bridge contests for international students, medical-related activities such as organizing blood donation events, and community and eldercare services. In addition, some participants engaged in university- and school-based volunteer services supporting events such as graduate thesis defenses and opening ceremonies, where they were responsible for tasks including coordination and communication, venue booking and setup, and minute-taking. This diversity of experiences ensured that the data captured perspectives from multiple volunteer contexts.

To ensure confidentiality and clarity in presenting the results, each participant was assigned an identifier (S1 to S15), with “S” representing “student”. These identifiers were used to refer to participants throughout the results and discussion sections.

This study was approved by the Association of Research Ethics Committees (Ethics Approval Number: AREC2025SFLL021102). Prior to the interview, all participants were provided with a detailed information sheet and signed an informed consent form, with the right to withdraw at any time without penalty. Data were collected, transcribed and analyzed under strict confidentiality and anonymity.

### 2.2. Research Instruments and Data Collection

Although this study aims to examine the factors that sustain university students’ participation in volunteering through the lens of Ecological Systems Theory (EST), the interview protocol did not include direct questions such as “What factors at the macrosystem level influence your volunteering?” Instead, participants were encouraged to recount their volunteering experiences and describe what volunteering meant to them in their own words. This approach was designed to elicit rich and authentic qualitative data. Although the EST framework was not explicitly introduced during the interviews, it guided the subsequent stages of coding and analysis. Using this indirect strategy enables volunteers’ motivations to emerge inductively rather than being shaped by predefined categories, thereby reducing response bias.

We employed semi-structured interviews to collect data. The interviews were designed to encourage participants to reflect on and share their volunteering experiences in depth. Specifically, participants were asked to (1) describe what being a volunteer meant to them, (2) recall memorable moments from their volunteering, (3) discuss experiences that motivated them to continue or, conversely, to withdraw from volunteering, (4) reflect on the initial moments or events that led them to become involved in volunteering, and (5) articulate what sustained their participation over time. These interviews followed a semi-structured format, with main questions providing a consistent framework while allowing the interviewer to ask follow-up or clarifying questions as needed to explore participants’ responses in depth.

We employed a snowball sampling method to identify potential participants. Initially, a small number of participants were contacted through university networks, who then referred other eligible individuals. All formal interviews were conducted online via mobile phone by the researcher, ranging from 15 to 20 min, and were audio-recorded with participants’ consent. To ensure comprehensive data collection, multiple rounds of interviews were carried out, with preliminary analysis conducted after each round to guide subsequent interviews. This iterative process continued until data saturation was achieved, with no new themes emerging, resulting in a three-month data collection period from June to September 2025.

### 2.3. Data Analysis, Reliability, and Validity

The interview data were analyzed following the six-phase framework for thematic analysis (Braun & Clarke, 2006), guided by Bronfenbrenner’s EST. A rolling analysis approach was adopted, where data collection and preliminary analysis occurred concurrently until data saturation was achieved.

Phase 1: Familiarizing with the data. All interview recordings were transcribed verbatim to create a textual dataset. We repeatedly read the transcripts, noting initial ideas and patterns.

Phase 2: Generating initial codes. The five systems of the Ecological Systems Theory served as an initial deductive framework. Significant statements or phrases from the transcripts were systematically extracted and given concise labels. This process remained open to emergent codes.

Phase 3: Searching for themes. The initial codes were collated and sorted into candidate themes. This involved clustering related codes to form coherent themes. For instance, codes such as peers influence and role models influence were integrated to form social influence. A thematic map was constructed to visualize the relationships between these candidate themes.

Phase 4: Reviewing themes. The candidate themes were rigorously reviewed in a two-stage process. First, all coded data within each theme were checked for coherence and consistency. Second, the entire thematic framework was reevaluated against the complete dataset to ensure it accurately reflected the breadth of the content. This iterative process led to the refinement, merging, or splitting of themes.

Phase 5: Defining and naming themes. Clear and precise names and definitions were developed for each refined theme. This phase involved identifying the core essence of each theme and its significance to the research question, culminating in a final thematic framework comprising 15 secondary dimensions.

Phase 6: Producing the report. The analysis was reported in the writing of the findings. The representative data extracts were selected to illustrate each theme, ensuring the report provides a truthful and insightful account of the factors influencing university students’ sustained participation in volunteering.

To ensure reliability of the coding process, two researchers independently coded randomly selected 20% of transcripts. Following the initial coding, they held a meeting to compare their codes, discuss discrepancies and refine the definitions of the codebook. Simultaneously, the coding framework was iteratively updated to align with emerging data (Corbin & Strauss, 1990). For example, we added peers influence to microsystem. This iterative process continued until a full agreement was reached on all coded segments. Then, the insights gained from this discussion were then used to refine the final codebook, which was subsequently applied by the primary researcher to the remaining transcripts.

Validity was enhanced through member checking, where participants reviewed the preliminary themes to confirm that their perspectives were accurately represented. Triangulation was also applied by cross-referencing participants’ demographic information and contextual data to support the interpretation of themes. This rigorous, systematic approach ensured the trustworthiness of the study’s findings.

## 3. Results

### 3.1. Microsystem Factors

In Bronfenbrenner’s Ecological Systems Theory (EST), the microsystem refers to the immediate settings in which individuals directly participate, such as home, school, workplace, etc. (Bronfenbrenner, 1977). The components of setting include place, time, physical features, activity, participant, and role. These settings constitute the most proximal layer of influence, shaping students’ attitudes, motivations, and behaviors through everyday interactions and relationships (Skinner et al., 2022). Although EST traditionally conceptualizes the microsystem as the immediate environments surrounding the individual, scholars also emphasize that individual characteristics, such as personal interest, self-efficacy, social interactions with others, etc., function as integral components of microsystem influences (Neal & Neal, 2013; Rosa & Tudge, 2013; Tudge et al., 2009). These characteristics shape how students interpret and respond to their immediate contexts, and thus directly affect their sustained participation in volunteering.

Drawing on the interview data, factors within the microsystem that influence university students’ sustained participation in volunteering can be categorized into the following four themes: personal growth need, facilitative personal attribute, social influence and environmental enabler (see Table 1).

#### 3.1.1. Facilitative Personal Attribute

Perceived competence. University students’ decisions to undertake volunteer tasks were significantly influenced by their perceived competence in relevant areas (*n* = 3). At the physical level, certain activities demanded extended hours or considerable stamina. Students who believed they possessed the necessary endurance were therefore more inclined to participate. For instance, S1 noted that her own stamina was relatively strong, making herself feel more qualified for tasks that might require staying up late or exerting substantial energy. At the psychological level, traits that students associated with being suitable for volunteer work also played a role. For example, S3 described himself as warm-hearted and enthusiastic, which made him more willing to take on service-oriented tasks. Likewise, S7 characterized herself as careful and composed, and these qualities she felt enabled her to handle responsibilities that required patience and stability.

Personal interest. For two participants, personal interest emerged as one of the powerful drivers of participation. These interests were closely tied to the nature of specific volunteer activities. Some students were motivated by opportunities to volunteer in environments that aligned with their personal interests or aesthetic preferences. For example, one participant described enjoying the chance to observe animals while volunteering at a zoo (S4), while another highlighted the appeal of coastal scenery during a beachside volunteer activity (S9). When the content of activity aligned with students’ personal preferences, their motivation to participate significantly increased.

#### 3.1.2. Personal Growth Need

Skill enhancement. Two of the interviewees emphasized that improving their abilities was a primary motivation for participating in volunteering. As an illustration, S1, a student majoring in English, noted that certain volunteer activities enabled direct interaction with international visitors. As a volunteer responsible for receiving foreign guests, S1 gained valuable opportunities to practice spoken English, which were perceived as relatively limited in regular coursework. Similarly, although S9 did not specify an academic major, the volunteer activities selected were closely related to the S9’s field of study, enabling the application of classroom knowledge in practical contexts. S1 also highlighted the development of organizational and coordination skills, explaining that coordinating volunteer events allowed her to observe real-world operations, gain hands-on experience, and improve her ability to manage activities. Although event planning and organization were not directly related to her major, she believed these skills would be beneficial for her future career, which may involve planning or coordinating tasks.

Cognitive development. Closely tied to skill enhancement was university students’ pursuit of cognitive growth. Two participants indicated that volunteering enabled them to encounter unfamiliar environments, social groups, or work settings, thereby broadening their understanding. S9 explicitly described this process as “expanding one’s horizons.” Likewise, S5 emphasized that volunteer work provided early exposure to workplace realities, enabling her to develop a more accurate understanding of the future career expectations. These experiences reflected volunteering’s role in enriching students’ cognitive frameworks and helping them make sense of the world beyond their immediate academic spheres.

Social interaction opportunity. Three of the participants identified opportunities for social interaction as an important motivator for participating in volunteer activities (*n* = 3). For some, volunteering served to strengthen existing relationships; as S2 explained, a core motivation was to deepen connections with roommates through shared experiences. For others, volunteering helped them build connections with a more diverse range of peers. S12, for example, saw volunteering as an opportunity to meet students from different majors, which naturally facilitated the development of a more diverse circle of friendships. Several participants highlighted that volunteering provided opportunities to interact more closely with teachers or engage with professionals from outside the university (e.g., S15), which they perceived as valuable experiences that supported their broader development during university life.

#### 3.1.3. Social Influence

Peers influence. Peers influence was mentioned by participants as a direct motivator, primarily through companionship and observational learning. The most common motivation was the desire for shared experience, as S5 explained that joining an activity with roommates built companionship bonds, where they encouraged each other to engage persistently in volunteer tasks and shared emotional feedback about their volunteering experiences. Beyond companionship, peers also served as a source of information that influenced individuals’ perceptions of volunteering’s intrinsic value. That means peers influence turned abstract ideas about volunteering value into real, personal experiences for individuals. S1, for instance, was influenced by a roommate who shared positive volunteering experiences. These accounts highlighted not only tangible benefits associated with the events (e.g., meals and snacks provided), but more importantly, the emotional rewards of volunteering, such as the gratification of being needed by the community and a tangible sense of self-efficacy derived from meaningful service. For a significant number of these students, their initial engagement was prompted directly by a peer’s invitation, with S5 noting she was persuaded by a close friend to her first event.

Role models influence. Inspiration from role models was a key factor for one participant, with motivation drawn from both public figures and personal acquaintances. For example, S4 reported that admiration for the renowned Guimei Zhang (an excellent Chinese teacher and principal of Huaping High School for Girls in Lijiang, Yunnan Province, who has dedicated to rural education) inspired the idea of participating in volunteer teaching programs in remote mountainous regions in China (i.e., regions with inadequate educational resources and infrastructure, predominantly located in inland provinces such as Yunnan, Guizhou, and Sichuan). Guimei Zhang’s dedication to education served as a specific example of meaningful volunteer work, motivating S4 to pursue teaching initiatives as a form of socially influential volunteering. The same participant (S4) also reported being profoundly influenced by an interview with a department head who built a volunteer organization, expressing a desire to engage more closely with such role models.

Family members influence. Family members influence was reported by one participant, typically establishing an early normative foundation for prosocial behavior, though its effect was not uniformly positive. In most cases, family members exerted a formative influence, with their own participation in volunteer activities serving as a direct stimulus for participants’ prosocial intentions. Specifically, S9 and S5 noted that their fathers’ regularly participation in community service set a good example for them, and S7 was similarly inspired to engage in volunteering by her uncle’s selfless voluntary work. However, a contrasting utilitarian perspective was reported by S13, whose family explicitly discouraged her participation, believing that as a woman, she should not waste time on activities without clear career benefit. This attitude reflected the conflict between traditional gender expectation and altruistic spirit of volunteerism, which may hinder individuals’ active participation in voluntary activities.

#### 3.1.4. Environmental Enabler

Pleasant atmosphere. A pleasant volunteer atomosphere was emphasized as a facilitator that enhance university students’ motivation for participating volunteer activities (*n* = 2). Actually, the pleasant volunteer atomosphere comes from two parts, tangible support and soft interpersonal atomosphere. For tangible support, essential supplies like meals and accommodation provided by the organizer avoided students’ daily concerns, letting them focus on volunteer tasks and making their whole service experience better, which made them more willing to participate in volunteer activities again (S8). For the soft interpersonal atomosphere, friendly and cooperative mood among volunteer peers made the activity process enjoyable. For example, S8 mentioned that the overall atomosphere of the volunteer department was particularly positive. Furthermore, S9 stated that working together toward a common goal made the volunteer work rewarding.

Positive feedback. Interview responses showed that positive feedback from those who benefited from the volunteer work was a strong driver (*n* = 2). S7 noted that the children she helped showed obvious gratitude and respect to volunteers, which made her feel happy and proud to do the work. Similarly, S9 shared that some parents of the children even returned to express thanks for their volunteer contributions, an act that made their hard work feel worthwhile. These positive feedback reinforced their commitment to volunteering, and also inspired them to advocate for volunteerism.

### 3.2. Exosystem and Macrosystem Factors

In Bronfenbrenner’s Ecological Systems Theory (EST), the exosystem refers to indirect social contexts that do not involve direct interaction with the individual but exert significant influence through their connections to microsystem or mesosystem. (Bronfenbrenner, 1977). These contexts typically include social institutions, workplace networks and media environments. The macrosystem differs from the other levels of context. It refers to the overarching institutional patterns of the culture or subculture, such as the economic, social, educational, legal, and political systems (Bronfenbrenner, 1977). It shapes the broader context for all other ecological systems. While the exosystem operates through indirect structural linkages, the macrosystem functions as a cultural framework that influences students’ perceptions of volunteering and their motivation to sustain participation.

Drawing on the interview data, factors within the exosystem and macrosystem that influence university students’ sustained participation in volunteering can be categorized into the following two themes: prosocial normative orientation and contextual enabler (see Table 2). Specifically, prosocial normative includes social contribution tendency and altruistic tendency. And contextual enabler comprises of organizational incentive and information accessibility.

#### 3.2.1. Prosocial Normative Orientation

Social contribution tendency. Social contribution tendency served as a specific motivator for one university students to take part in volunteer activities with national attributes. Certain students tended to choose volunteer work related to public interests and national needs because they believed such activities have universal social value. For example, S3 said he was more willing to join volunteer activities like flood relief and pandemic prevention, which are closely linked to national and social well-being. The statement showed that social contribution tendency made students more inclined to engage in volunteer activities with social significance.

Altruistic tendency. Altruistic tendency was another important factor for university students’ volunteer participation (*n* = 4). It means students get a sense of fulfillment from helping others. For example, S6 shared that teaching peers first aid skills in campus training brought meaningful because it could save people’s lives in emergencies, which strengthened the sustained determination to participate in volunteering. Furthermore, S7 mentioned that planning a charity sale meant dealing with a lot of trivial tasks like arranging stalls, coordinating participants and managing supplies, which left her physically and mentally tired. However, knowing that the sale would support people in remote and poor areas made the experience deeply meaningful, and she willing to engage in this altruistic work. Additionally, S4 said stepping out of her comfort zone to help others brought happiness and a sense of achievement. These examples proved that students’ desire to help others and gain emotional satisfaction from volunteer activities are powerful drivers for their engagement, even when the work is tiring or tedious.

#### 3.2.2. Contextual Enabler

Organizational incentive. Organizational incentive also motivated university students to participate in volunteer activities (*n* = 3). These incentives are tangible rewards that make students’ efforts recognized. S4 said volunteer participation can earn points for scholarships. Additionally, there were also monetary rewards for outstanding volunteers (S5). S8 added that participants could get participation certificates as a recognition of their volunteer work. These rewards not only gave students material or honorary returns but also fostered a sense of recognition for their contributions, thus increasing their willingness to sustain volunteering.

Information accessibility. Information accessibility reduced barriers for university students to participate in volunteering (*n* = 2). With the popularity of social media, students can quickly get updates on volunteer activities. S10 mentioned that volunteer activity information was frequently updated on WeChat, a widely used social media platform in China. S9 also said community groups (grassroots organizations formed by local residents to serve their neighborhood) were important channels to get volunteer activity notices. Convenient information channels let students know about volunteer opportunities in a timely manner, making it easier for them to choose and participate in activities that suit them.

### 3.3. Chronosystem Factors

The chronosystem encompasses change or consistency over time not only in the characteristics of the person but also of the environments in which the person lives (Bronfenbrenner, 1979, 1994). In this study, the influence of the chronosystem is primarily reflected in participants’ accounts of large-scale historical events, one key dimension of this system.

Two participants explicitly described how large-scale historical events shaped their intentions to participate in volunteer activities. To illustrate, S7 noted that during the COVID-19 pandemic, extensive media coverage and public discussion about frontline volunteers significantly shaped their perceptions. As S7 explained, the severe societal impact of the pandemic, “made me feel that volunteers are truly admirable, and made me want to participate in related volunteer activities immediately.” This suggests that salient historical events can play a role in activating individuals’ inclination for volunteering by elevating the perceived social value, urgency, and moral significance of volunteer work.

Notably, no themes emerged in the interview data relating to individual trajectories (e.g., age-related changes in goals or capabilities) or temporal changes in immediate contexts (e.g., shifts in personal life responsibilities), which are also components of the chronosystem. The absence of these themes warrants further examination, as the reasons remain unclear.

### 3.4. Restrictive Factors

Data revealed that university students’ sustained participation in volunteering is not only driven by positive factors but also constrained by multiple-level constraints, which can be categorized into internal factors and external factors, as detailed below:

#### 3.4.1. Internal Restrictive Factors

University students’ willingness to participate in volunteering was shaped by their subjective experience of activities. Some students were reluctant to take part in volunteering alone, finding the process tedious without peer company (S2). Additionally, tedious tasks such as long hours of standing (S6) and extreme physical and mental fatigue from early mornings and late nights (S11) prompted students to avoid relevant volunteer activities. Some even quit volunteering entirely when the workload exceeded their personal capacity to cope (S5), highlighting how negative experiential factors directly decreased their participation intention.

Students’ cost–benefit evaluation of volunteering also acted as a key constraint. They consciously weighed the time and energy invested in volunteer work against the tangible and intangible returns received. If students perceived rewards such as subsidies or a sense of achievement as inadequate to offset their efforts (S5), or if financial subsidies were so low that they failed to cover even basic expenses (S8), they would directly choose not to participate in volunteer activities. This cognitive assessment reflected a rational decision-making process among students when considering volunteer participation.

#### 3.4.2. External Restrictive Factors

External restrictive factors hindering university students’ volunteer participation mainly included three aspects: information barriers, limited recruitment opportunities, and practical logistical challenges.

Information barriers emerged as a primary restrictive factor for university students’ volunteer participation. Unlike the positive role of accessible information channels in boosting participation (discussed in Result Section 3.2), students in this study faced various obstacles in obtaining valid volunteer information. First, formal information channels were lacking: some students expressed a willingness to participate in specific activities (e.g., caring for orphans, S4) but failed to find official registration channels or reliable sources of information. Second, information transmission was untimely: S15 reported hearing about volunteer activities only after the registration period had closed, and the delay in information transmission directly resulted in missed participation opportunities. Third, information coverage was limited: volunteer information was often spread only through peer networks (e.g., S14 learned about nucleic acid tests volunteering from classmates) rather than official university platforms, leading to uneven access to information among students. These information barriers directly obstruct students to engage in volunteer activities, even when they had strong participation intentions.

Limited recruitment opportunities also restricted students’ access to volunteer activities. On the one hand, recruitment quotas for popular volunteer activities were limited (e.g., Spring Festival Gala volunteers, S1), and the recruitment scope of some activities was restricted to universities in specific regions (e.g., universities in Chengdu, S15). Additionally, some activities set strict selection criteria such as requiring award-winning experiences (S8), which directly excluded some students. On the other hand, practical challenges became significant obstacles to students’ participation in volunteering. Time conflicts were a common issue: S10 noted that their part-time job and academic schedule often conflicted with volunteer activities’ time, leaving them unable to participate. Commuting also posed a problem. Specifically, S8 stated that the one-way commute to volunteer place took two to three hours, and round-trip time required four to six hours. Moreover, external disruptions such as pandemic-related mobility restrictions (S14) further limited students to engage in volunteer work. Overall, these external factors created significant barriers to students’ participation in voluntary work, underscoring the need for more accessible and inclusive volunteering initiatives.

## 4. Discussion

Drawing on in-depth interviews with 15 Chinese university students, this study explored the factors that shape and sustain university students’ long-term participation in volunteering. The findings showed that university students’ volunteering is not influenced by isolated factors, but by diverse factors ranging from individual attributes to broader societal and temporal contexts.

Employing the Ecological Systems Theory (EST) as an analytical framework proved particularly advantageous, as it enabled us to comprehensively identify a range of factors influencing volunteer participation. Compared with previous studies that explored the influencing factors of volunteer participation and yielded relatively simplistic conclusions, some focused solely on microsystem and exosystem factors, such as employability, skills and enhanced learning (Holdsworth, 2010), and organizational factors affecting volunteers collectively (Studer & Von Schnurbein, 2013). To illustrate, we classified the organizational incentive and information accessibility into exosystem and macrosystem factors, which influenced individuals’ sustained participation in volunteering.

Additionally, a distinctive finding from our research is the role of the chronosystem, one of the core components of EST. One participant stated being motivated to volunteer after seeing pandemic volunteers’ stories. As a significant historical event, COVID-19 profoundly reshaped environments at all levels: it simultaneously stimulated individual altruism, promoted the establishment of community mutual-aid networks, and influenced government policies concerning public health. This holistic perspective, made possible by EST, provides a more comprehensive explanation of sustained volunteer participation.

Despite its strengths, applying EST in volunteer participation analysis also posed challenges, which can be seen in both the inherent complexity of EST and the practical applicability of findings derived from it.

First, the EST is characterized by a high level of conceptual complexity. As (Darling, 2007) noted, the propositions of Bronfenbrenner’s EST are inherently complex, reflecting the multilayered and interdependent systems they seek to represent. The specification of multiple systems, each with its own conceptual definitions and illustrative examples, contributes to theoretical richness but introduces substantial analytical complexity. In applied analysis, this level of complexity markedly complicates efforts to identify and interpret the factors shaping a particular behavior, given that influences frequently span multiple ecological levels.

Second, using EST as the analytical framework for thematic analysis also limits the practical applicability of findings. For example, Sheerin et al. (2023) proposed the application of EST to selecting outcomes to assess intervention effects in juvenile legal system (JLS) intervention research to better capture proximal and distal influences on youth behavior. But their focus on constructing a measurement framework for categorizing social-ecological domains left limited room for practical discussion. Specifically, little attention was paid to how these categorized factors could be translated into actionable, real-world intervention strategies. This emphasis on theoretical framework creates an analytical burden, underscoring the need for conceptual tools that help researchers navigate EST’s breadth while maintaining the clarity required to apply findings in practice.

Building on these observations, we introduce controllability as an additional analytical lens for understanding the factors that shape students’ volunteer participation. This concept highlights that motivational determinants vary in the extent to which they can be acted upon by relevant agents, including students themselves as well as external actors such as universities and non-governmental organizations. Accordingly, we propose classifying factors influencing volunteer participation into three categories: controllable, partially controllable, and minimally controllable.

Controllable factors are those that the agent (e.g., student, university, non-profit organization) can directly modify or regulate through deliberate action. For example, from the students’ perspective, personal interests constitute a controllable factor, as they reflect preferences and motivational orientations that can be consciously adjusted within a relatively short time frame, thereby directly shaping volunteer behavior.

Partially controllable factors cannot be fully determined by the agent but can be meaningfully influenced through deliberate choices, communication, or sustained socialization. These typically include social and environmental influences within the microsystem. For instance, although students cannot control the behaviors of peers or family members, they can choose whether to accept peer invitations or draw inspiration from family members and role models, as mentioned in interviews with S4 and S7. Similarly, while students cannot directly construct a positive organizational climate as short-term volunteers, they can choose to join institutions with a supportive work environment. Likewise, although beneficiaries’ expressions of appreciation are not under the students’ direct control, students can increase the likelihood of receiving positive feedback by actively engaging with them and demonstrating commitment during volunteering. This category also encompasses traits shaped by long-term socialization, such as altruistic tendencies, which are difficult to change rapidly but can be gradually influenced over time.

Minimally controllable factors reflect structural or contextual conditions that the agent can do little to alter. Examples include organizational incentive imposed by universities or organizations and broader historical or macro-level factors. These factors shape the context within which motivation unfolds but remain largely beyond students’ direct influence.

The categorization of factors into three levels of controllability, namely controllable, partially controllable, and minimally controllable, is theoretically associated with attribution theory and perceived behavioral control (PBC). Attribution theory refers to the study of the perception or inference of cause (Kelley & Michela, 1980). It provides the framework necessary to understand how individuals explain the causes of events in their environment happened (Martinko & Mackey, 2019). For example, when students or organizations attribute volunteering-related outcomes to internal and controllable factors, they are more likely to perceive control over their participation. Furthermore, perceived behavioral control refers to the extent to which the behaviour is under voluntary control, the ease or difficulty of performing the behaviour (Trafimow et al., 2002). That means individuals are more likely to form a behavioral intention when they perceive high control over the target behavior (Hagger et al., 2022). These two theories provide useful lenses for analyzing university students’ sustained participation in volunteering. The former theory explains how individuals interpret the causes of their volunteer participation outcomes (i.e., success or difficulty in sustaining volunteer work) by attributing theses outcomes to controllable or uncontrollable factors. This process, in turn, shapes their willingness to sustain volunteering. The latter theory helps predict whether individuals will form an intention to sustain their volunteer participation by assessing their perceived ability to control the behaviors required for volunteering (e.g., task difficulty, time commitment). Thus, these theoretical lenses help us figure out how different agents shape students’ volunteer participation.

In considering sustaining students’ participation with volunteering, it is important to recognize that the agent is not necessarily the students themselves, but the agents responsible for fostering their motivation, such as universities, volunteer organizations, and program coordinators. The “controllable, partially controllable, minimally controllable” framework remains relevant in this broader context, although some differences emerge. For instance, organizational incentive, considered minimally controllable from the students’ perspective, become fully controllable for the institutions implementing them. Conversely, personal interest, fully controllable by students themselves, may be regarded as partially controllable from the perspective of an external agent seeking to influence motivation.

Notably, the controllability of factors dynamically changes across an individual’s life stages. As a case in point, in this study, S4, as a university student, needed to earn volunteer service points for scholarship. However, once S4 graduates, she no longer need to apply for scholarship; as a result, this specific incentive will ceases to be a motivating factor. This example indicates that certain minimally controllable factors from the students’ perspective are not static but shift with life stages. It reminds us that static analysis is insufficient when examining factors influencing volunteer participation; instead, we should analyze how these factors change over time, thereby more comprehensively revealing the factors affecting university students’ sustained volunteer participation.

The primary value of the proposed “controllable, partially controllable, minimally controllable” framework lies in its ability to move beyond identifying the elements that influence individual behavior toward clarifying what can be done, by whom, and to what extent. By explicitly distinguishing degrees of controllability and specifying the relevant agents, the framework enhances the practical feasibility of volunteer motivation cultivation. It enables universities, volunteer organizations, and program coordinators to identify actionable leverage points rather than treating all influencing factors as equally amenable to intervention. In doing so, the framework strengthens the translation of analytical insights into feasible, agent-sensitive strategies for sustaining volunteer participation. Moreover, by foregrounding agency and differential controllability, this approach mitigates a common limitation of highly comprehensive models, namely their tendency to generate analytically rich but practically diffuse findings, thereby offering a clearer pathway from explanation to intervention.

Notably, the analysis of chronosystem-related factors indicates that the study’s findings may be biased. According to the EST, the chronosystem refers to change over time not only in the characteristics of the person but also of the environments in which the person lives (Bronfenbrenner, 1979, 1994). These changes include not only historical events, but also within-person developments such as evolving values and capabilities, as well as temporal shifts in immediate contexts, such as fluctuations in family or academic circumstances. However, the interview responses in this study overwhelmingly concentrated on the first type, namely historical events such as COVID-19, while offering limited insight into the latter two dimensions. As a result, chronosystem influences are only partially captured in this study.

One possible explanation is that chronosystem influences are, by nature, gradual, implicit, and difficult for individuals to recognize. For instance, participants rarely explicitly notice changes such as the maturation of their values or shifts in their capabilities over time. Even when such changes occur, individuals are unlikely to articulate them using abstract temporal language, such as “growth” and “longitudinal change”, when reporting their experiences in an interview. Consequently, these influences are often underreported in interview narratives.

Moreover, the influence of chronosystem factors does not stem from isolated events, but rather from the cumulative, delayed, and nonlinear effects of experiences over time. These processes, such as gradually developing a sense of social responsibility or becoming more capable and confident with age, are often perceived as natural, taken-for-granted developments rather than explicit drivers of volunteer motivation. Participants rarely recognize these changes as discrete key events, and as a result, they are seldom reported as chronosystem influences, which limits their visibility in qualitative data.

To better capture chronosystem influences, future research could incorporate explicit temporal prompts in interviews, adopt longitudinal or repeated-interview designs, or triangulate multiple data sources such as reflective journals or participation records. Analytical frameworks that emphasize temporal or developmental processes, such as life-course perspectives, can further help identify gradual, cumulative, and context-dependent effects. These strategies can enhance the detection of subtle chronosystem influences and provide a more comprehensive understanding of the factors shaping one’s behaviors or behavioral intention, such as university students’ volunteering and their motivation to participate.

## 5. Conclusions

This study employed a qualitative research design, conducting semi-structured interviews with 15 university students to investigate the factors influencing their sustained participation in volunteering. Through a thematic analysis guided by Bronfenbrenner’s Ecological Systems Theory (EST), a total of 15 sub-dimensions of influencing factors were identified across the microsystem, exosystem, macrosystem and chronosystem, which answers the three research questions.

Beyond mapping these multi-level influences, the study placed particular emphasis on introducing controllability as a complementary analytical lens to address the practical challenges posed by highly comprehensive frameworks. In addition, special attention was devoted to the chronosystem, highlighting potential analytical biases associated with static interpretations of motivational factors and underscoring the value of a temporal perspective for understanding constraints and opportunities specific to each life stage.

University administrators and volunteer coordinators can use these findings to design programs that better support students’ sustained participation by addressing factors at multiple levels and over time. Policy makers and educators can also apply these insights to create interventions that target controllable factors, making volunteer engagement more effective and sustainable.

One limitation of this study is its reliance on self-reported interview data, which may be susceptible to social desirability bias, whereby participants report socially desirable responses rather than their genuine thoughts or behaviors. Future research may address this limitation by adopting observational methods or longitudinal designs to more objectively capture behavioral patterns.

In addition, as the primary focus of this study was on factors driving participation in volunteering, relatively less attention was devoted to exploring inhibiting or constraining factors. Although participants were invited to reflect on barriers to volunteering, fewer responses were provided in this regard. As a result, the present findings offer a more comprehensive understanding of motivational drivers than of potential obstacles. Future studies may adopt a more balanced design to further investigate the interplay between facilitating and hindering factors.

In addition, this study was conducted with a sample of Chinese university students, primarily due to accessibility considerations. Although the findings offer insights into factors influencing sustained volunteering participation, the results may reflect characteristics specific to the sociocultural and educational context in China. Therefore, caution should be exercised when generalizing the findings to other national or cultural settings. Future research could examine whether the identified factors operate similarly across different cultural contexts.

Future research could advance this field through longitudinal investigations of sustained volunteer participation among university students, offering insight into the developmental and maintenance processes of volunteer motivation. Researchers could also evaluate the effectiveness of specific intervention strategies, thereby facilitating the translation of research findings into practical applications.

## Figures and Tables

**Table 1:** Microsystem Factors that Influence University Students’ Sustained Participation in Volunteering.

Main Category	Subcategory	Original Statement
facilitative personal attribute	perceived competence	S1: I have great stamina. Volunteering sometimes is labour-intensive, and I feel capable of handling it.
		S3: I’m a warm-hearted and enthusiastic person. That’s why I’m so motivated to help others and get involved in volunteering.
		S7: I’m very careful and composed (which I believe helps me with volunteering tasks).
	personal interest	S4: It (volunteering at the zoo) is interesting because I can see the animals.
		S9: I can take in the ocean view and the scenery while volunteering at the beach.
personal growth need	skill enhancement	S1: I can interact with foreigners during volunteering. I may practice my spoken English.
		S9: The volunteer activity I choose is related to my major. I can apply the theories from my major to real-world problems through the volunteering.
	cognitive development	S9: Participating in such activities broadens my horizons.
		S5: It enables me to master key job processes and better navigate the realities of today’s job market.
	social interaction opportunity	S2: Volunteering with roommates is a great way to strengthen our relationships.
		S12: I can meet peers from different majors and make friends with them.
		S15: Volunteering enables me to connect with a wide range of people and even engage in meaningful conversations with teachers.
social influence	peers influence	S5: When my roommates decide to participate in a volunteer activity, I want to join them as well.
		S5: A close friend encouraged me to join a campus volunteer activity, and that was how I got started.
	role models influence	S4: I want to volunteer as a teacher in remote villages, inspired by Principal Guimei Zhang. I hope to continue her mission of serving through education.
		S4: … Seeing his dedication made me feel that such individuals are truly admirable, and it inspired me to move closer to that kind of example.
	family members influence	S7: My uncle performs unpaid acts of service. Seeing his selflessness motivates me to consider participating in volunteer activities.
environmental enabler	pleasant atmosphere	S8: The atmosphere is very positive.
		S9: I feel working with them together on a task is pleasant and rewarding.
	positive feedback	S7: I feel that children showed deep appreciation and respect for volunteers. Therefore, I feel happy and honored.
		S9: Some of the parents may come back to thank me for my efforts.

**Table 2:** Exosystem and Macrosystem Factors that Influence University Students’ Sustained Participation in Volunteering.

Main Category	Subcategory	Original Statement
prosocial normative orientation	social contribution tendency	S3: I was more inclined to participate in volunteer activities with national attributes, like flood relief or pandemic volunteering.
	altruistic tendency	S3: … such activities carried universal, public welfare value.
		S3: I thought tree-planting is meaningful for public health and natural environment.
		S6: When I taught peers first aid skills in campus training, I found it meaningful to save individual’s lives in emergencies. Therefore, I always signed up for such activities.
		S7: I helped plan a charity sale, which was tiring but could help in remote and poor areas.
		S4: Stepping out of my comfort zone to help others brought me happiness and pride.
contextual enabler	organizational incentive	S4: Points for scholarship.
		S5: Monetary rewards.
		S8: Participation certificates.
	information accessibility	S10: Frequent updates on Wechat (a popular social media platform in China)
		S9: Community groups (grassroots organizations formed by local residents to serve their neighborhood)

## Data Availability

The data are not available due to ethical restrictions.
